# Cyclopyranil (Pesticides)

**DOI:** 10.14252/foodsafetyfscj.D-26-00002

**Published:** 2026-03-19

**Authors:** Food Safety Commission of Japan

## Abstract

Food Safety Commission of Japan (FSCJ) conducted a risk assessment of cyclopyranil (CAS No. 1651191-47-7), a pyrazolylpyrazole herbicide, based on results from submitted documents. The data used in the assessment include fate in plants (paddy rice), residues in crops, fate in animals (rats), subacute toxicity (rats, mice and dogs), chronic toxicity (dogs), combined chronic toxicity/carcinogenicity (rats), carcinogenicity (mice), two-generation reproductive toxicity (rats), developmental toxicity (rats and rabbits) and genotoxicity. Major adverse effects of cyclopyranil were observed in body weight (suppressed weight gain), the liver (effects including organ weight increases and hepatocellular vacuolation), the kidney (effects including lipofuscin deposition in renal tubules), and the brain (cerebral neuropil and white matter vacuolation in dogs) (**Table 1**). Adverse effects were observed on neither fertility, teratogenicity, nor genotoxicity. The lowest NOAEL for potential adverse effects after a single oral administration of cyclopyranil was 60 mg/kg bw per day from the result of a developmental toxicity study in rabbits (**Table 2**). FSCJ specified an acute reference dose (ARfD) of 0.6 mg/kg bw by applying a safety factor of 100 to this NOAEL.

## Conclusion in Brief

Food Safety Commission of Japan (FSCJ) conducted a risk assessment of cyclopyranil (CAS No. 1651191-47-7), a pyrazolylpyrazole herbicide, based on results from submitted documents.

The data used in the assessment include fate in plants (paddy rice), residues in crops, fate in animals (rats), subacute toxicity (rats, mice and dogs), chronic toxicity (dogs), combined chronic toxicity/carcinogenicity (rats), carcinogenicity (mice), two-generation reproductive toxicity (rats), developmental toxicity (rats and rabbits) and genotoxicity.

Major adverse effects of cyclopyranil were observed in body weight (suppressed weight gain), the liver (effects including organ weight increases and hepatocellular vacuolation), the kidney (effects including lipofuscin deposition in renal tubules), and the brain (cerebral neuropil and white matter vacuolation in dogs) (Table 1). Adverse effects were observed on neither fertility, teratogenicity, nor genotoxicity.

**Table 1.** *Levels relevant to toxicological evaluation of cyclopyranil*

Species	Study	Dose (mg/kg bw per day)	NOAEL (mg/kg bw per day)	LOAEL (mg/kg bw per day)	Critical endpoints^1^^)^
Rat	90-day subacute toxicity study	0, 200, 1 300, 8 500, 20 000 ppm	M: 76.4 F: 88.9	M: 509 F: 560	M/F: Suppressed body weight gain, etc.
		M: 0, 12.0, 76.4, 509, 1 160 F: 0, 14.5, 88.9, 560, 1 320			
	Two-year combined chronic toxicity/ carcinogenicity study	0, 200, 2 000, 6 000, 20 000/10 000 ppm	M: 8.22 F: 10.1	M: 81.8 F: 103	M/F: Suppressed body weight gain, lipofuscin deposition in renal tubules, etc. (M: Increased incidences of testicular interstitial cell tumors, islet cell adenoma, and adenocarcinoma F: Increased incidence of uterine horn adenocarcinoma
		Carcinogenicity study group: M: 0, 8.22, 81.8, 246, 842 F: 0, 10.1, 103, 326, 878 Chronic toxicity study group: M: 9.20, 94.9, 278, 939 F: 11.9, 118, 364, 1 160			
	Two-generation reproductive toxicity study	0, 125, 250, 500 ppm	Parents PM: 29.4 PF: 35.1 F_1_M: 36.6 F_1_F: 41.3 Offspring PM: 15.0 PF: 17.4 F_1_M: 18.5 F_1_F: 21.1	Parents PM: - PF: - F_1_M: - F_1_F: - Offspring PM: 29.4 PF: 35.1 F_1_M: 36.6 F_1_F: 41.3	Parents: No toxicity Offspring: Low body weight (No effect on fertility was observed)
		PM: 0, 7.36, 15.0, 29.4 PF: 0, 8.9, 17.4, 35.1 F_1_M: 0, 9.12, 18.5, 36.6 F_1_F: 0, 10.3, 21.1, 41.3			
	Developmental toxicity study	0, 3, 6, 15, 50	Dams: 50 Fetuses: 6	Dams: - Fetuses: 15	Dams: No toxicity Fetuses: Low body weight (No teratogenicity was observed)
Mouse	90-day subacute toxicity study	0, 100, 1 000, 7 000 ppm	M: 13.0 F: 152	M: 131 F: 1 130	M: Basophilic change of renal tubules F: Increased reticulocyte counts, etc.
		M: 0, 13.0, 131, 881 F: 0, 15.5, 152, 1 130			
	78-week carcinogenicity study	0, 70, 700, 2 800, 7 000 ppm	M: 7.74 F: 7.39	M: 81.1 F: 76.6	M/F: Lipofuscin deposition in renal tubules, etc. (M: Increased incidence of hepatocellular adenomas)
		M: 0, 7.74, 81.1, 324, 799 F: 0, 7.39, 76.6, 314, 808			
Rabbit	Developmental toxicity study	0, 12, 60, 300	Dams: 12 Fetuses: 12	Dams: 60 Fetuses: 60	Dams: Suppressed body weight gain Fetuses: Skeletal variations (supernumerary ribs) (No teratogenicity observed)
Dog	90-day subacute toxicity study	0, 400, 4 000, 40 000/20 000/8 000 ppm	M: 12.1 F: 13.6	M: 118 F: 133	M/F: Vacuolation of cerebral neuropil and white matter, etc.
		M: 0, 12.1, 118, 306 F: 0, 13.6, 133, 345			
	One-year chronic toxicity study	0, 70, 240, 850, 3 000 ppm	M: 6.90 F: 25.4	M: 23.8 F: 87.9	M: Increases in total cholesterol F: Vacuolation of cerebral neuropil and white matter, etc.
		M: 0, 1.96, 6.90, 23.8, 84.5 F: 0, 2.10, 7.34, 25.4, 87.9			
ADI			NOAEL: 6 SF: 100 ADI: 0.06		
The critical study for setting ADI			Developmental toxicity study (dog)		

ADI, Acceptable daily intake; NOAEL, No-observed-adverse-effect level; SF, Safety factor

-: LOAEL could not be specified.

^1)^ The adverse effect observed at LOAEL

In a two-year combined chronic toxicity/carcinogenicity study in rats, a significant increase in the incidence of testicular interstitial cell tumors was observed, along with trends toward increased incidences of pancreatic islet cell adenoma and adenocarcinoma in males. A significant increase in the frequency of uterine horn adenocarcinoma was also observed in females. In a 78-week carcinogenicity study in mice, a significant increase was observed in the incidence of hepatocellular adenoma in males. The mode of action was considered to be non-genotoxic, and thus a threshold upon evaluation was possible to be established.

Based on results of related experiments, cyclopyranil (parent compound only) was identified as the substance relevant for the residue definition for dietary risk assessment in agricultural and fishery products.

The lowest no-observed-adverse-effect level (NOAEL) was 6 mg/kg bw per day from a study of developmental toxicity in rats among the studies examined. FSCJ specified an acceptable daily intake (ADI) of 0.06 mg/kg bw per day by applying a safety factor of 100 to the NOAEL.

The lowest NOAEL for potential adverse effects after a single oral administration of cyclopyranil was 60 mg/kg bw per day from the result of a developmental toxicity study in rabbits (Table 2). FSCJ specified an acute reference dose (ARfD) of 0.6 mg/kg bw by applying a safety factor of 100 to this NOAEL.

**Table 2.** *Potential adverse effects of a single oral administration of cyclopyranil*

Species	Study	Dose (mg/kg bw)	Endpoints relevant to setting NOAEL and ARfD (mg/kg bw per day)^1^^)^
Rabbit	Developmental toxicity study	0, 12, 60, 300	Dams: 60 Dams: Decreased body weight, decreased food intake
Dog	90-day subacute toxicity study	0, 400, 4 000, 40 000/ 20 000/8 000 ppm	M: 118 F: 133 M/F: Vomiting
		M: 0, 12.1, 118, 306 F: 0, 13.6, 133, 345	
ARfD			NOAEL: 60 SF: 100 ARfD: 0.6
The critical study for setting ARfD			Developmental toxicity study (rabbit)

ARfD, Acute reference dose; NOAEL, No-observed-adverse-effect level; SF: Safety factor

^1)^ The adverse effect observed at LOAEL

## References

[1] Food Safety Commission of Japan. Risk Assessment Report. Cyclopyranil (Pesticides) [in Japanese]. https://www.fsc.go.jp/fsciis/attachedFile/download?retrievalId=kya20240612080&fileId=210.

